# Supplementary material for: An integrated proteome and transcriptome of B cell maturation defines poised activation states of transitional and mature B cells
Source: Nat Commun. 2023 Aug 23;14:5116. doi: 10.1038/s41467-023-40621-2 (PMC10447577; doi:10.1038/s41467-023-40621-2)
Supplement: Supplementary file 3 — Description of Additional Supplementary Files [file 41467_2023_40621_MOESM3_ESM.pdf]

Supplementary Data Legends:

Supplementary Data 1: a, Proteomics of T1, T2, MZ and FoB cells containing peptide counts, copy numbers per cell and LFQ intensities. **b**, Differentially expressed proteins that are found in all B cell subsets. **c**, Differentially expressed proteins that are unique to T1 and MZ B cells.

Supplementary Data 2: Transcript per million (TPM) and protein copy numbers of genes identified by Illumina RNA-sequencing and proteomics.

Supplementary Data 3: a, Early activation genes extracted from DESeq analysis of Garruss et al. (ref. 56). **b**, PC-related genes extracted from DESeq analysis of Gaudette et al. (ref. 16).

Supplementary Data 4: Full-length transcript measured by Oxford Nanopore Technology (ONT) sequencing as in Fig S5b and compared to Illumina sequencing.

Supplementary Data 5: a, List of poised mRNAs linked to early activation in B cells. **b**, List of poised mRNAs linked to plasmablast formation and antibody secretion.

Supplementary Data 6: List of antibodies used for flow cytometry analysis.
