# Supplementary material for: An integrated proteome and transcriptome of B cell maturation defines poised activation states of transitional and mature B cells
Source: Nat Commun. 2023 Aug 23;14:5116. doi: 10.1038/s41467-023-40621-2 (PMC10447577; doi:10.1038/s41467-023-40621-2)
Supplement: Supplementary file 10 — Reporting Summary [file 41467_2023_40621_MOESM10_ESM.pdf]

Corresponding author(s): Salerno & Turner

Last updated by author(s): Jul 07, 2023

Reporting Summary

Nature Portfolio wishes to improve the reproducibility of the work that we publish. This form provides structure for consistency and transparency in reporting. For further information on Nature Portfolio policies, see our Editorial Policies and the Editorial Policy Checklist.

Please do not complete any field with "not applicable" or n/a. Refer to the help text for what text to use if an item is not relevant to your study.
For final submission: please carefully check your responses for accuracy; you will not be able to make changes later.

Statistics

For all statistical analyses, confirm that the following items are present in the figure legend, table legend, main text, or Methods section.

n/a	Confirmed	
☐	☒	The exact sample size (*n*) for each experimental group/condition, given as a discrete number and unit of measurement
☐	☒	A statement on whether measurements were taken from distinct samples or whether the same sample was measured repeatedly
☐	☒	The statistical test(s) used AND whether they are one- or two-sided<br>*Only common tests should be described solely by name; describe more complex techniques in the Methods section.*
☐	☒	A description of all covariates tested
☐	☒	A description of any assumptions or corrections, such as tests of normality and adjustment for multiple comparisons
☐	☒	A full description of the statistical parameters including central tendency (e.g. means) or other basic estimates (e.g. regression coefficient) AND variation (e.g. standard deviation) or associated estimates of uncertainty (e.g. confidence intervals)
☐	☒	For null hypothesis testing, the test statistic (e.g. *F*, *t*, *r*) with confidence intervals, effect sizes, degrees of freedom and *P* value noted<br>*Give P values as exact values whenever suitable.*
☒	☐	For Bayesian analysis, information on the choice of priors and Markov chain Monte Carlo settings
☐	☒	For hierarchical and complex designs, identification of the appropriate level for tests and full reporting of outcomes
☐	☒	Estimates of effect sizes (e.g. Cohen's *d*, Pearson's *r*), indicating how they were calculated

Our web collection on statistics for biologists contains articles on many of the points above.

Software and code

Policy information about availability of computer code

Data collection

Mass Spectrometry: Orbitrap Q Exactive Plus mass spectrometer (Thermo Scientific)
Illumina RNA-sequencing: Illumina HiSeq 2500-RapidRun system on a 50bp single-end mode with a coverage of 20M reads per sample
Oxford Nanopore Technologies (ONT) sequencing: MinION R9.4.1 flow cell using cDNA PCR sequencing (SQK-LSK109) with a coverage of 26.5M total reads

Data analysis

Proteomics:
- MaxQuant software package (1.6.10.43)
- Perseus (1.6.10.45)
- Ensembl BioMart (2.46.3)
- R (4.0)
- ggplot2 (3.3.3)
- VennDiagram CRAN package (1.6.20)
- pHeatmap (1.0.12)
- GraphPad Prism 9

Transcriptomics:
- FastQC (0.11.9; http://www.bioinformatics.babraham.ac.uk/projects/fastqc/)
- Trim Galore (https://www.bioinformatics.babraham.ac.uk/projects/trim_galore/)
- HiSat2 (2.1.0)
- SeqMonk (1.47.2; https://www.bioinformatics.babraham.ac.uk/projects/seqmonk/)
- StrigTie (2.1.1)

- DESeq2 (1.30.1)
- R (4.0)
- Guppy (4.0.11)
- Pychopper (2.5.0)
- Minimap2 (2.17-r941)
- Samtools (1.9)
- Integrative Genomics Viewer (IGV 2.7.2)
- GraphPad Prism 9

For manuscripts utilizing custom algorithms or software that are central to the research but not yet described in published literature, software must be made available to editors and reviewers. We strongly encourage code deposition in a community repository (e.g. GitHub). See the Nature Portfolio guidelines for submitting code & software for further information.

Data

Policy information about availability of data

All manuscripts must include a data availability statement. This statement should provide the following information, where applicable:

- Accession codes, unique identifiers, or web links for publicly available datasets
- A description of any restrictions on data availability
- For clinical datasets or third party data, please ensure that the statement adheres to our policy

All data generated and analysed during this study are included in this article and its supplementary information, or have been made available in public repositories. Raw mass spectrometry data files, MaxQuant analysis files and fasta database files are available from the ProteomeXchange data repository (https://proteomecentral.proteomexchange.org/cgi/GetDataset). The proteomic data of T1, T2, MZ and FoB cells are accessible with the identifier PXD043349, whereas the proteomic data of WT and PDCD4 KO MZ B cells are accessible with the identifier PXD043351. Analysed proteomics data used to generate figures are available in Supplementary Data 1a-c. Both Illumina and ONT sequencing data generated in this study are available from the NCBI Gene Expression Omnibus (GEO) repository under the accession code GSE178728 (https://www.ncbi.nlm.nih.gov/geo/query/acc.cgi?acc=GSE178728). Calculated TPMs and protein copy numbers of genes identified by Illumina sequencing and proteomics are reported in Supplementary Data 2. Lists of early activation genes and PB-related genes extracted upon DESeq2 analysis of RNA-sequencing from refs.16, 56 are reported in Supplementary Data 3a-b. A side-by-side comparison of TPMs of genes that were detected by both ONT and Illumina sequencing is reported in Supplementary Data 4. A list of poised mRNAs in B cells is provided in Supplementary Data 5a-b. Flow cytometry data that support the finding of this study are available from the authors upon request. The raw numbers for graphs are available in the Source Data file.

Human research participants

Policy information about studies involving human research participants and Sex and Gender in Research.

Reporting on sex and gender	n.a.
Population characteristics	n.a.
Recruitment	n.a.
Ethics oversight	n.a.

Note that full information on the approval of the study protocol must also be provided in the manuscript.

Field-specific reporting

Please select the one below that is the best fit for your research. If you are not sure, read the appropriate sections before making your selection.

☒ Life sciences ☐ Behavioural & social sciences ☐ Ecological, evolutionary & environmental sciences

For a reference copy of the document with all sections, see nature.com/documents/nr-reporting-summary-flat.pdf

Life sciences study design

All studies must disclose on these points even when the disclosure is negative.

Sample size	Mass spectrometry and RNA-seq experiments include 3 or 4 biological replicates per group.<br>For in vivo mouse experiments, sample size was determined for each experiment with a priori power analysis performed by G*power plug-in software, as required and approved by the Babraham Institute Animal Welfare and Ethical Review Body, and in compliance with existing European Union and United Kingdom Home Office legislation and local standards.
Data exclusions	No data were excluded when analysing proteomics and transcriptomics datasets<br>For in vivo mouse experiments, all analyzed mice were included for analysis.
Replication	All data were reliably reproduced, and compiled from independent experiments in figures and statistical analysis as indicated in the figure

March 2021

Replication	legends.
Randomization	To ensure that B cell subsets isolated from the same mouse were analysed by Illumina sequencing, ONT sequencing and proteomics, randomization of mice was not applicable to paired proteomic and transcriptomic analysis.<br>For in vivo mouse experiments, mice receiving donor BM cells of different origins were randomly co-house in the same cage.
Blinding	Mass spectrometry and RNA-seq samples were number coded prior to analysis.<br>For in vivo mouse experiments, donor BM cells of different origins were number coded prior to injection.<br>No further blinding was applied in this study, as internal controls were used in each experiment to obtain unbiased data.

Reporting for specific materials, systems and methods

We require information from authors about some types of materials, experimental systems and methods used in many studies. Here, indicate whether each material, system or method listed is relevant to your study. If you are not sure if a list item applies to your research, read the appropriate section before selecting a response.

Materials & experimental systems

n/a	Involved in the study	
☐	☒	Antibodies
☒	☐	Eukaryotic cell lines
☒	☐	Palaeontology and archaeology
☐	☒	Animals and other organisms
☒	☐	Clinical data
☒	☐	Dual use research of concern

Methods

n/a	Involved in the study	
☒	☐	ChIP-seq
☐	☒	Flow cytometry
☒	☐	MRI-based neuroimaging

Antibodies

Antibodies used

Name / Clone name / Fluorochrome / Catalogue no. / (most used) Lot no. / dilution factor / manufacturer

anti-B220 /RA3-6B2 / BUV395 / 563793 / 0121784 / 1:300 / BD Horizon
anti-B220 /RA3-6B2 / FITC / 35-0452-U500 / C0452012417354 / 1:300 / TONBO Biosciences
anti-CD1D / 1B1 / AF647 / #123511 / B246235 / 1:100 / Biolegend
anti-CD4 / RM4-5 / FITC / 100510 / B214387/ 1:400 / Biolegend
anti-CD8 / 53-6.7 / FITC / 100706 / B217242/ 1:400 / Biolegend
anti-CD8 / 53-6.7 / APC / 17-0081-82 / 2023410 / 1:400 / Invitrogen
anti-CD19 / 6D5 / FITC / 11-0193-82 / 2002716 / 1:400 / Invitrogen
anti-CD19 / 1D3 / PerCP-Cy5.5 / 152406 / B255189 / 1:400 / Biolegend
anti-CD19 / 1D3 / BUV737 / 612781 / B0287656 / 1:400 / BD Horizon
anti-CD21 / 7G6 / PE / 552957 / 3273981 / 1:400 / BD Pharmingen
anti-CD21 / 7G6 / FITC / 561769 / 3345726 / 1:200 / BD Pharmingen
anti-CD22 / OX-97 / APC / 126109 / B217708 / 1:200 / Biolegend
anti-CD23 / B3B4 / BV421 / 101621 / B209223 / 1:400 / Biolegend
anti-CD23 / B3B4 / PE / 553139 / 4251668 / 1:400 / BD Pharmingen
anti-CD40 / 1C10 / APC / 17-0401-81 / 1995461 / 1:200 / Invitrogen
anti-CD45.1 / A20 / BUV395 / 565212 / 0279550 / 1:200 / BD Horizon
anti-CD45.1 / A20 / BV421 / 110732 / B263122 / 1:400 / Biolegend
anti-CD45.2 / 104 / BUV737 / 612779 / 9297856 / 1:200 / BD Horizon
anti-CD45.2 / 104 / BV786 / 109839 / B334349 / 1:400 / Biolegend
anti-CD93 / AA4.1 / APC / 17-5892-83 / E029491 / 1:100 / eBioscience
anti-CD93 / AA4.1 / BV421 / 747716 / 0164358 / 1:100 / BD OptiBuild
anti-CD138 / 281-2 / BV786 / 740880 / 0268196 / 1:200 / BD OptiBuild
anti-CD180 / RP/14 / biotin / 552159 / 0000045653 / 1:200 / BD
anti-CD267/TACI / 8F10-3 / PE / 12-5942-81 / 2045475 / 1:300 / Invitrogen
anti-IgD / 11-26c.2a / BV510 / 405723 / B214952 / 1:600 / Biolegend
anti-IgD / 11-26c.2a / AF488 / 405718 / B245377 / 1:600 / Biolegend
anti-IgM / II/41 / PeCy7 / 25-5790-81 / 2039912 / 1:400 / Invitrogen
anti-IgM / RMM-1 / APC / 406509 / B138285 / 1:200 / Biolegend
anti-IgG3 / R40-82 / BV421 / 565808 / 7338642 / 1:100 / BD Horizon
anti-Ly6C/G / RB6-8C5 / PE / 553128 / 2118821 / 1:400 / BD Pharmingen
anti-eIF4A1 / rabbit polyclonal antibody / unconjugated / ab31217 / 1:200 / Abcam
anti-PDCD4 / rabbit monoclonal antibody / unconjugated / ab79405 / 1:200 / Abcam
anti-Puromycin / 12D10 / AF647 / MABE343-AF647 / 1:100 / Sigma-Aldrich
Streptavidin / BV510 / 405234 / B258255 / 1:600 / Biolegend
Streptavidin / PeCy7 / 405206 / B278254 / 1:1800 / Biolegend
Secodary donkey anti-rabbit IgG(H+L) antibody / AF647 / 711-605-152 / 153705 / 1:600 / Jackson

NP-specific cells were detected with 4-Hydroxy-3-iodo-5-nitrophenylacetic acid NIP(15)-BSA-biotin (1:8000 dilution factor / N1027-5 / Biosearch Technologies)

	Dead cells were excluded using fixable cell viability dye eF780 (Thermofisher / 1:2000 dilution factor)<br>All cells were blocked with FcgR(CD16/32)-blocking antibody (2.4G2 / BioXcell)
Validation	We have validated the specificity of the rabbit polyclonal anti-eIF4A1 antibody and the rabbit monoclonal anti-PDCD4 antibody using eIF4A1 KO and PDCD4 KO mice, respectively. We have confirmed the specificity of the anti-Puromycin antibody using mice that were not injected with puromycin, and were therefore considered as a negative control. For all the other primary antibodies we have relied on validation provided by supplier.

Animals and other research organisms

Policy information about studies involving animals; ARRIVE guidelines recommended for reporting animal research, and Sex and Gender in Research

Laboratory animals	For paired proteomics and transcriptomics, 12-week-old male and female C57BL/6 mice were used.<br>For in vivo mouse experiments, 8 to 12-week-old male B6.SJL-PtprcaPepcb/BoyJ mice were used as recipient mice. Donor BM cells were prepared from 9 to 10-week-old male C57BL/6, B6.129S6-Pdcd4tm1Yhcn/J, B6.SJL-PtprcaPepcb/BoyJ and B6.SJL-Ighmtm1Cgn mice.
Wild animals	No wild mice were used in this study
Reporting on sex	For paired proteomics and transcriptomics, B cell substes were sorted from male mice (indicated as biological replicate sample number 1-2-4 = BR1, BR2, BR4) and female mice (indicated as biological replicate sample number 3 = BR3).<br>All in vivo mouse experiment were performed using male mice, due to mice availability.
Field-collected samples	No field collected samples were used for this study
Ethics oversight	All mouse experiments were approved by the Babraham Institute Animal Welfare and Ethical Review Body, and complied with existing European Union and United Kingdom Home Office legislation and local standards.

Note that full information on the approval of the study protocol must also be provided in the manuscript.

Flow Cytometry

Plots

Confirm that:

☒ The axis labels state the marker and fluorochrome used (e.g. CD4-FITC).

☒ The axis scales are clearly visible. Include numbers along axes only for bottom left plot of group (a 'group' is an analysis of identical markers).

☒ All plots are contour plots with outliers or pseudocolor plots.

☒ A numerical value for number of cells or percentage (with statistics) is provided.

Methodology

Sample preparation	Single cell suspension was achieved by meshing mouse spleens over a 70uM strainer and washing with Dutch modified RPMI-1640 containing 10% FBS, 100X glutamax (cat. n. 35050-061), 100U/ml penicilin and 100ug/ml streptomycin (cat. n. 15140-122), and 50uM 2-mercaptoethanol (cat. n. 31350-010; all Gibco). Red blood cells were lysed using ACK lysis buffer (A10492-01; Gibco).<br>For cell surface staining, single cell suspensions were prepared in PBS supplemented with 1% FBS and 2mM EDTA.<br>For intracellular staining, cells were fixed and permeabilized using the CytoFix/CytoPerm kit (cat. n. 51-2090KZ; 51-2091KZ; BD Biosciences)
Instrument	LSR Fortessa Flow Cytometer equipped with 355nm, 405nm, 488nm, 561nm and 640nm lasers (BD Biosciences)<br>FACSAria III equipped with 405nm, 488nm, 561nm and 640nm lasers (Becton Dickinson)<br>FACSAria Fusion equipped with 355nm, 405nm, 488nm, 561nm and 640nm lasers (Becton Dickinson)
Software	FlowJo software (TreeStar, version 10.6.1); GraphPad Prism 9
Cell population abundance	Cell sorting was performed using a 70uM nozzle and sorters were set on 4-way purity mode. The purity of the post-sort cell fractions was > 99.9% in all samples and determined by flow cytometry analysis.
Gating strategy	Full gating strategies are provided in Figure S9. For all datasets, lymphocytes were selected using forward and side scatter properties and doublet exclusion. Dead cells were excluded from the analysis by using the fixable cell viability dye eF780 (Thermofisher).

☒ Tick this box to confirm that a figure exemplifying the gating strategy is provided in the Supplementary Information.

This checklist template is licensed under a Creative Commons Attribution 4.0 International License, which permits use, sharing, adaptation, distribution and reproduction in any medium or format, as long as you give appropriate credit to the original author(s) and the source, provide a link to the Creative Commons license, and indicate if changes were made. The images or other third party material in this article are included in the article's Creative Commons license, unless indicated otherwise in a credit line to the material. If material is not included in the article's Creative Commons license and your intended use is not permitted by statutory regulation or exceeds the permitted use, you will need to obtain permission directly from the copyright holder. To view a copy of this license, visit http://creativecommons.org/licenses/by/4.0/
